# High spin current density in gate-tunable spin-valves based on graphene nanoribbons

**DOI:** 10.1038/s41598-023-36478-6

**Published:** 2023-06-07

**Authors:** Chun-Pu Wang, Shih-Hung Cheng, Wen-Jeng Hsueh

**Affiliations:** Nanomagnetism Group, Department of Engineering Science and Ocean Engineering, National Taiwan University, 1, Sec. 4, Roosevelt Road, Taipei, 10617 Taiwan

**Keywords:** Materials science, Nanoscience and technology

## Abstract

The usage of two-dimensional (2D) materials will be very advantageous for many developing spintronic device designs, providing a superior method of managing spin. Non-volatile memory technologies, particularly magnetic random-access memories (MRAMs), characterized by 2D materials are the goal of the effort. A sufficiently large spin current density is indispensable for the writing mode of MRAMs to switch states. How to attain spin current density beyond critical values around 5 MA/cm^2^ in 2D materials at room temperature is the greatest obstacle to overcome. Here, we first theoretically propose a spin valve based on graphene nanoribbons (GNRs) to generate a huge spin current density at room temperature. The spin current density can achieve the critical value with the help of tunable gate voltage. The highest spin current density can reach 15 MA/cm^2^ by adjusting the band gap energy of GNRs and exchange strength in our proposed gate-tunable spin-valve. Also, ultralow writing power can be obtained, successfully overcoming the difficulties traditional magnetic tunnel junction-based MRAMs have faced. Furthermore, the proposed spin-valve meets the reading mode criteria and the MR ratios are always higher than 100%. These results may open the feasibility avenues for spin logic devices based on 2D materials.

## Introduction

Spintronics has played an important role in overcoming the limitations of traditional technologies and attracted great attention for decades. The use of two-dimensional (2D) materials has lately allowed researchers to do previously unthinkable experiments and test conceptual frameworks of spintronics owing to their ultrathin thickness and unique physical properties^1–3^. Therefore, a growing number of spintronic devices based on 2D materials, such as graphene^3,4^, transition metal dichalcogenides (TMDs)^5^, and topological insulators (TIs)^6^, have been demonstrated more recently.

Magnetic random-access memory (MRAM) is one promising spintronic device, suited for high-efficiency computation and edge computing used in AI, IoT, and machine learning^7,8^. Besides, MRAM has sparked considerable attention due to its non-volatility and high read/write performance, making it an appealing replacement for DRAM, SRAM, and Flash^9,10^. Despite being prospective, traditional magnetic tunnel junction (MTJ)-based MRAMs still have some defects. For instance, spin-transfer torque MRAMs (STT-MRAMs) suffer from disadvantages such as high switching power and insufficient endurance^11^. Additionally, scaling down the size of MTJs, needing an additional magnetic field, and requiring high switching power are weaknesses for spin–orbit torque MRAMs (SOT-MRAMs)^12,13^. To avoid aforesaid drawbacks, current research has concentrated on 2D-based magnetic memory technologies^14,15^. The creation, injection, detection, transmission, and manipulation of the spin signal are the main factors that impact the reading and writing performances in the 2D-based magnetic memory^16,17^.

Reading and writing are two quite important functions of MRAMs, characterized by magnetoresistance (MR) ratio and spin current density, respectively. A minimum MR ratio of around 20% is required to read the state in MRAM technologies^18^. MR ratios of 0.73%^19^ and 5%^20^ were reported experimentally based on 2D materials. Researchers have found that the MR ratio of 2D-based spin-valves may meet the applicable requirement for reading in theoretical prediction^21–23^. On the other hand, to write the state, critical spin current density (CSCD) of around 5 MA/cm^2^ at room temperature is required to switch between two states of the free layer in the memory^24–26^. It’s essential to maintain the thermal stability, which forbids lowering the excessive CSCD in practical use^26^. Therefore, how to generate spin current density beyond the critical value is a vital issue for designing 2D-based spin valves^27^.

Spin current induced switching could be understood in terms of spin-transfer torque effect^25,26^, while it was first experimentally demonstrated in a graphene-based spin valve by Lin et al.^28,29^, revealing a spin current density around 2 MA/cm^2^ can be obtained. However, applying an external magnetic field and operating under a relatively low temperature were both needed in their experiments^28,29^. As far as we know, there is currently no research reporting that huge spin current density can be obtained in graphene at room temperature.

The gate-tunable spin-valve based on armchair graphene nanoribbon (AGNR) to produce a significant STT effect is theoretically proposed in this study, allowing us to switch the magnetization without the aid of an external magnetic field at room temperature. It is discovered that without applying a gate voltage, the spin current density is about 1.5 MA/cm^2^, which does not exceed the CSCD. With the help of a tunable gate, a huge spin current density of around 15 MA/cm^2^ can be reached by modulating the band structure, which greatly surpassed the CSCD. Furthermore, the ultralow writing power is attainable in the proposed spin-valve. The reading performance, MR ratio, of our device can exceed 100% at a relatively low bias to meet the applicable requirement. The STT and MR effect influenced by various physical parameters, including bias voltage, band gap energy, and exchange splitting energy will be discussed in this simulation work as well.

## Results and discussion

**Figure 1:**
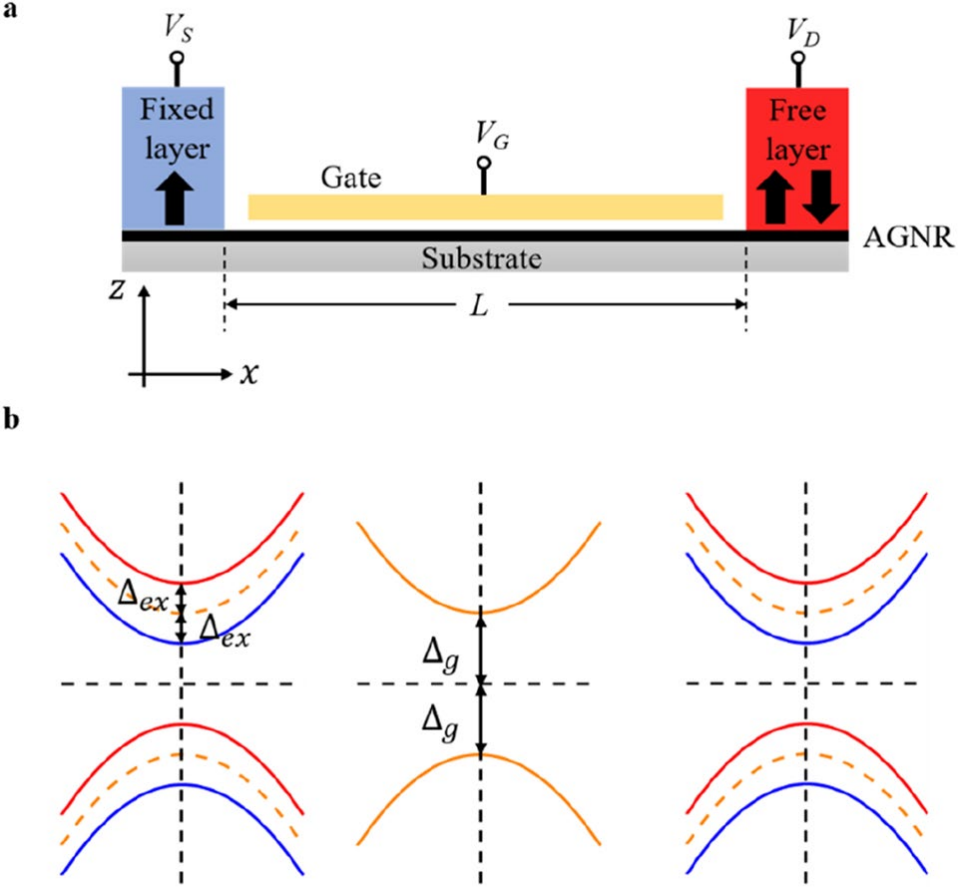
Gate-tunable spin-valve based on armchair graphene nanoribbons. (**a**) Schematic illustration and device profile of the spin-valve based on armchair graphene nanoribbons with tunable top gate. Considering the out-of-plane setup, two ferromagnetic leads—the fixed layer (blue) and the free layer (red)—will generate the exchange field. (**b**) Band structure for the states in two ferromagnetic leads (left and right) and channel (middle). The exchange splitting energy and the bandgap energy are denoted by ${\Delta }_{ex}$ and ${\Delta }_{g}$ respectively.

In this study, an AGNR-based spin valve with a tunable top gate is considered, as sketched in Fig. 1a. The monolayer nanoribbon, where the current flows through, is taken to be the *x–y* plane, and the out-of-plane setup is taken into account. As specified the source and drain, respectively, are the fixed layer (blue) and the free layer (red), which are ferromagnets with magnetization in the *z*-direction. Both the fixed layer and the free layer have lengths of 10 nm. As shown in Fig. 1b, the ferromagnet on the AGNR channel will cause a magnetic proximity effect, which results in exchange splitting in the band structure. Yang et al. demonstrated that graphene possesses an exchange splitting energy 36 meV via the interaction between graphene and EuO^30^. Furthermore, Wu et al. demonstrated that monolayer graphene may be magnetized by CrSe, with an exchange splitting energy of 67 meV^31^. As a result, we vary the magnitude of the exchange splitting energy in the appropriate range of 20 to 80 meV in this study, and the band structure of spin-down electrons and spin-up electrons are denoted by the red curve and blue curve in Fig. 1b respectively. Only the band gap energy restricted by the edge state will affect the transport characteristics in the region with the top gate^32–34^, and the channel length is set to 10 nm in the scaling limit, which prevents current leakage^35^. The Fermi velocity is set to 10^6^ m/s. Our calculations are performed at a temperature 300 K.

**Figure 2:**
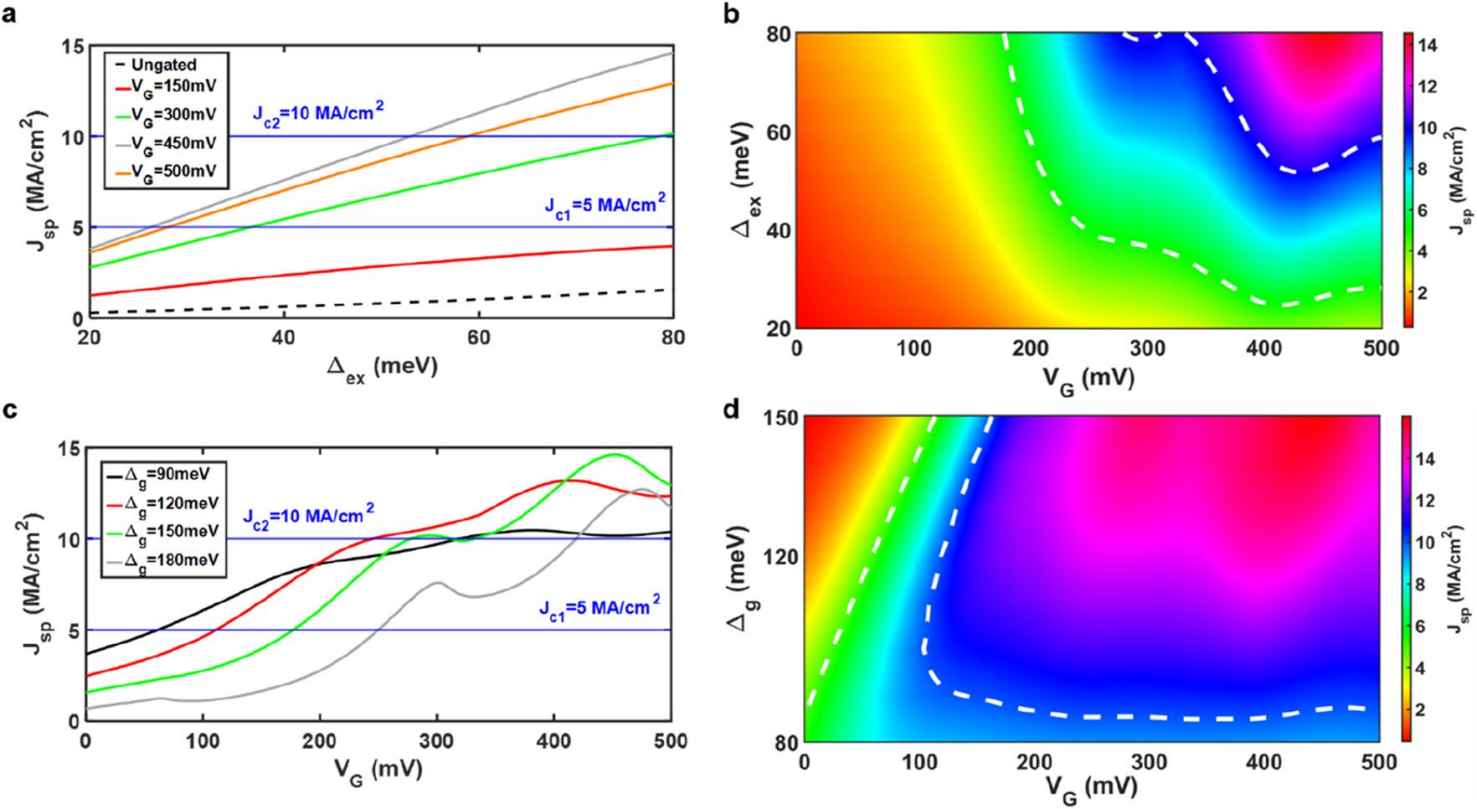
High spin current density in gate-tunable spin-valves based on armchair graphene nanoribbons. (**a**) Spin current density versus exchange splitting energy with different gate voltages. (**b**) Contour colormap concerning exchange splitting energy and gate voltage. The band gap energy is set to be 150 meV in (**a**) and (**b**). (**c**) Spin current density as a function of gate voltage with different band gap energy. Two blue lines in both (**a**) and (**c**) indicate the critical values for switching states. Note that the spin current density in the ungated spin-valve (black dashed line) cannot surpass the minimum critical value always, as shown in (**a**). (**d**) Contour colormap for band gap energy and gate voltage. Likewise, white dashed lines in (**b**) and (**d**) indicate the critical values for switching. The exchange splitting energy is 80 meV in both (**c**) and (**d**), while bias voltage 0.5 V is applied for these cases.

The STT and MR effects are two crucial phenomena when discussing spin-dependent transport properties in MRAMs and spin-valves. The STT is proportional to spin current density^24,25^, while a large enough spin current density is required to flip the magnetization of the free layer. High spin current density is achieved with the help of applied gate voltages in the proposed structure, as shown in Fig. 2. In writing mode, the applied bias voltage *V*_*SD*_ is usually set to be 0.5 V, which is adopted for calculations in Fig. 2. In Fig. 2a, it is shown that spin current density *J*_*sp*_ enlarges as the exchange splitting energy ${\Delta }_{ex}$ increases. The maximum spin current density *J*_*sp*_ is around 15 MA/cm^2^ for ${\Delta }_{ex}$ = 80 meV when the gate voltage *V*_*G*_ is 450 mV. If the gate voltage *V*_*G*_ is tuned up to be 500 mV, the spin current density *J*_*sp*_ will decrease. Also, it can be observed that the spin current density can even not exceed the minimum CSCD 5 MA/cm^2^ when *V*_*G*_ is 150 and 0 mV (ungated), as indicated by the red solid line and black dashed line in Fig. 2a, respectively. Contour colormap for spin current density *J*_*sp*_ with respect to exchange splitting energy ${\Delta }_{ex}$ and gate voltage *V*_*G*_ is shown in Fig. 2b, which allows us to visualize the operating region for switching clearly. It is suggested that the operating gate voltage *V*_*G*_ > 200 mV and exchange splitting energy ${\Delta }_{ex}$ > 40 meV, while two white dashed lines stand for critical switching values. The optimal region is around ${\Delta }_{ex}$ = 80 meV and *V*_*G*_ = 450 mV, which is consistent with the result in Fig. 2a.

To find the optimal case again, the exchange splitting energy ${\Delta }_{ex}$ is set to be 80 meV in Fig. 2c,d. The band gap energy ${\Delta }_{g}$ is proportional to 1/*W*, where *W* is the width of AGNR, as depicted in Fig. 1b^32–34^. Thus, the band gap energy ${\Delta }_{g}$ can be adjusted, which is in the range of 90 to 180 meV in our cases. The relations between the spin current density and the gate voltage with different band gap energy are shown in Fig. 2c. When the gate voltage is less than 200 mV, it is observable that the spin current density increases with decreasing band gap energy. It can be seen that all of them surpass the minimum critical value 5 MA/cm^2^ when the gate voltage *V*_*G*_ is larger than 250 mV. Besides, the maximum spin current density *J*_*sp*_ reaches 14 MA/cm^2^ when the band gap energy ${\Delta }_{g}$ equals 150 meV and the gate voltage *V*_*G*_ is around 450 mV. Again, to realize the spin current density *J*_*sp*_ more precisely regarding the band gap energy ${\Delta }_{g}$ and the gate voltage *V*_*G*_, the contour colormap is illustrated in Fig. 2d. The hilltop is located at ${\Delta }_{g}$ = 150 meV and *V*_*G*_ = 450 mV, which is consistent with the result in Fig. 2c. Note that the spin current density is steady no matter how the channel length varies (see Supplementary Note 4 for details).

**Figure 3:**
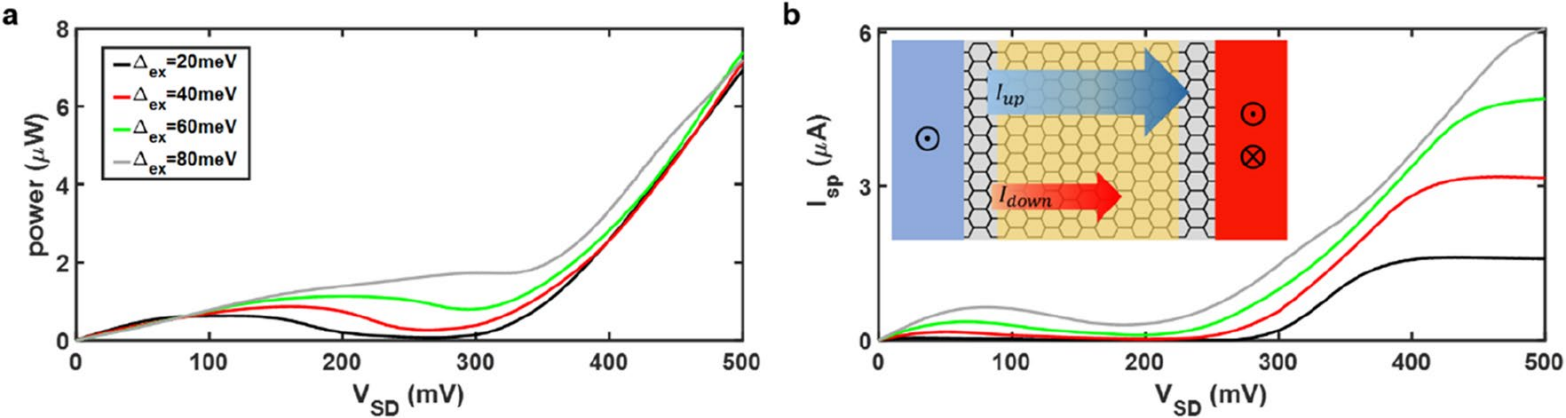
Power and spin current in gated spin-valves. (**a**) Power and (**b**) spin current as a function of bias voltage with different exchange splitting energy. The inset in (**b**) demonstrates that the spin-up electrons are the majority carriers transmitting, and the spin current ${I}_{sp}$ is defined as ${I}_{up}-{I}_{down}$. In (**a**) and (**b**), the band gap energy is set to 150 meV, while gate voltage 450 mV is applied.

In order to further understand the performance of the AGNR spin-valve, the power consumption and the spin current are presented in Fig. 3. In Fig. 3a, it is shown that the power consumption will have the global maximum at *V*_*SD*_ = 500 mV. The negative differential resistance effect can be observed in the interval of local maximum and local minimum. Besides, the power consumptions for all cases are almost the same when operating at *V*_*SD*_ = 500 mV (writing voltage). Ultralow writing power can be obtained in the proposed spin-valve. In Fig. 3b, the spin current *I*_*sp*_ as a function of bias voltage *V*_*SD*_ is exhibited. The scheme in Fig. 3b demonstrates the spin-up electrons are the majority carriers. It is found that the spin current *I*_*sp*_ becomes larger as the exchange splitting energy increases. Interestingly, when operating at writing voltage, the spin current at ${\Delta }_{ex}$= 80 meV (maximum) is 4 times larger than that at ${\Delta }_{ex}$ = 20 meV (minimum). Therefore, it is suggested that the exchange splitting energy may be as larger as possible, which can lead to enlarged STT and polarization for switching and will nearly not consume extra energy at the same bias voltage (see Supplementary Note 2 for details).

**Figure 4:**
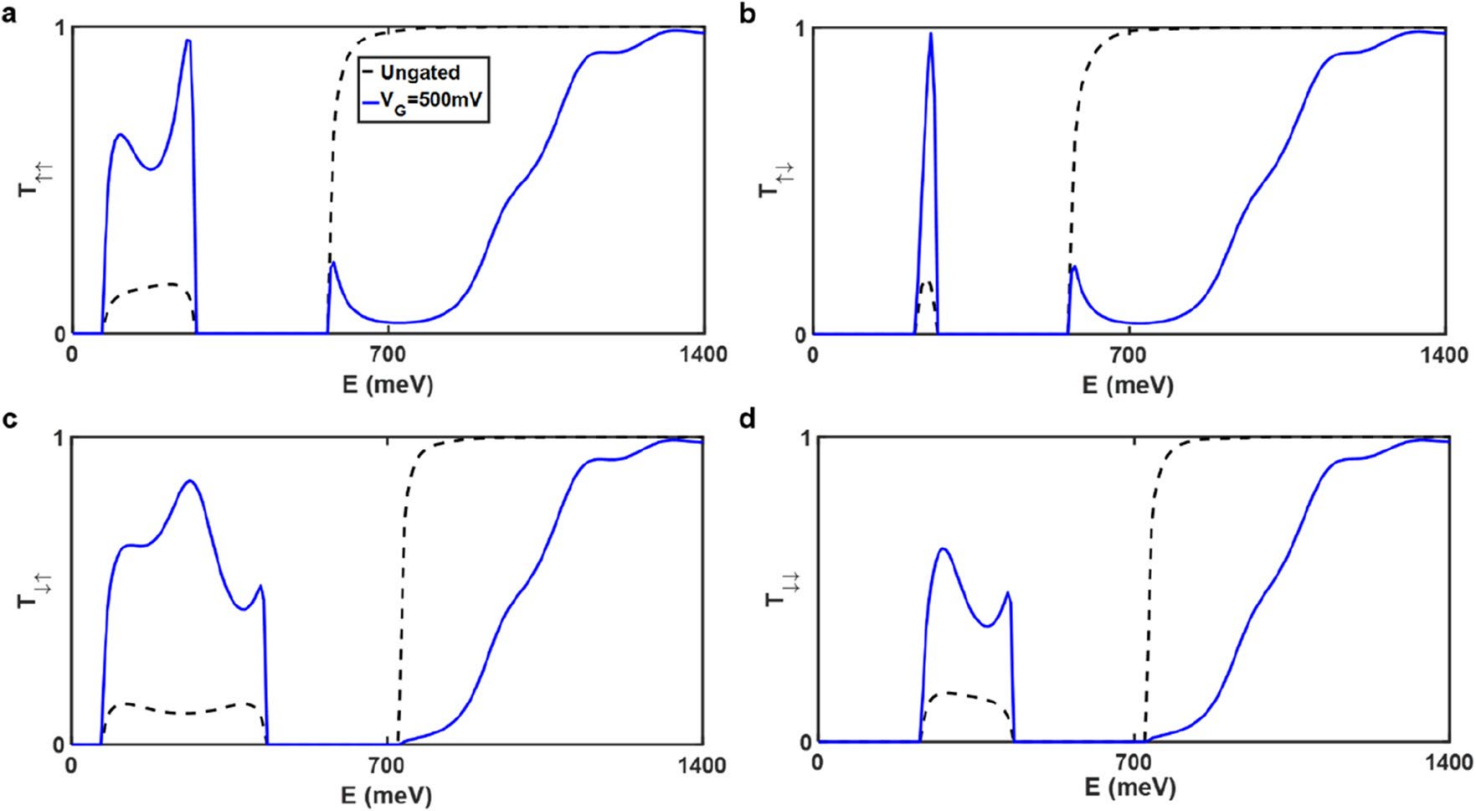
Spin-dependent transmission function versus electron energy. (**a**) *T*_*↑↑*_, (**b**) *T*_*↑↓*_, (**c**) *T*_*↓↑*_, and (**d**) *T*_*↓↓*_ versus electron energy in ungated (black dashed line) and gated (blue solid line) spin-valves. Transmission functions *T*_*ij*_ are depicted as a function of electron energy. The subscript *i* (*j*) denotes the spin orientation, while ↑ and ↓ represent spin-up and spin-down states, respectively. The calculations are performed with bias voltage 500 mV, band gap energy 150 meV, and exchange splitting energy 80 meV.

For a better understanding of the STT performances in gated and ungated AGNR spin-valves, the spin-dependent transmissions are illustrated in Fig. 4. The parameters are given as follows: the bias voltage *V*_*SD*_ = 500 mV, the band gap energy ${\Delta }_{g}$ = 150 meV, and the exchange splitting energy ${\Delta }_{ex}$ = 80 meV. The blue solid line indicates the gated spin-valve, while the black dashed line represents the ungated spin-valve. In Fig. 4, it can be observed that there are two forbidden energy bands in each panel. The intervals of them are influenced by the exchange splitting energy when considering the same width of AGNR. The net contribution to spin current density *J*_*sp*_ is the spin-up contributions (Fig. 4a,c) minus the spin-down contributions (Fig. 4b,d). It is shown that the transmission ${T}_{ij}$ in the integrand of Eq. (2) becomes larger in the relatively low energy window, which results in an enlarged STT effect in the gated AGNR spin-valve (See Methods Section for details). The gate voltage limits the transmission during the high energy window. The transmission in Fig. 4a (4c) and Fig. 4b (4d), whether in gated or ungated spin-valves, is essentially equal in the relatively high energy domain, effectively canceling the contribution to the spin current and resulting in the generation of just charge current. The charge current will be reduced as a result of the gate voltage being applied due to restricted transmission. To produce a significant STT effect and lower power consumption in the proposed spin-valve, gate voltage must be applied.

**Figure 5:**
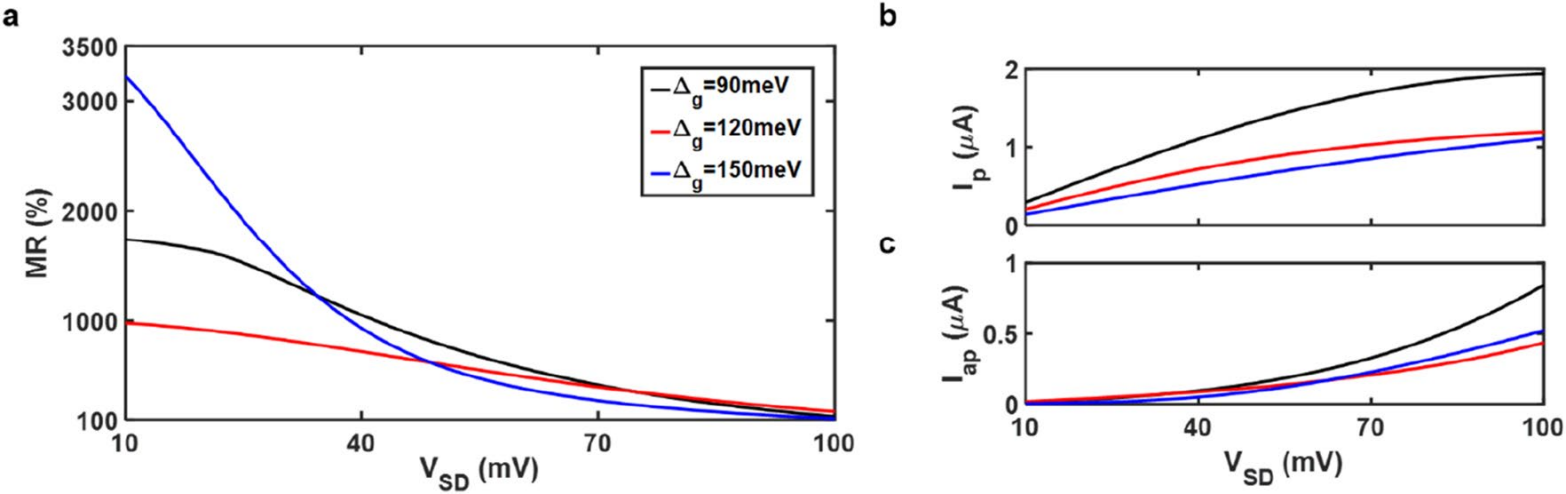
MR ratio versus bias voltage in gated spin-valves. (**a**) MR ratio versus a relatively small sensing bias with different band gap energy. Note that the applied bias voltage is around 0.1 V for reading. Spin-polarized current in the (**b**) parallel and **c** antiparallel configurations are plotted to investigate the MR effect in (**a**). The exchange splitting energy is set to be 80 meV, and a gate voltage 450 mV is applied.

This study also examines reading performance. In the suggested spin-valve, a relatively small sensing bias is used to read the state as depicted in Fig. 5. To characterize the reading performance, we introduce the MR ratio, MR = $\frac{{I}_{p}-{I}_{ap}}{{I}_{ap}}\times 100\%$, where ${I}_{p}$ and ${I}_{ap}$ are the spin-polarized currents in the parallel and antiparallel configurations respectively. Figure 5a displays the bias-dependent MR ratio for various band gap energies. The maximum MR ratio always occurs at bias voltage *V*_*SD*_ = 10 mV, and it is about 3200% for band gap energy ${\Delta }_{g}$ = 150 meV. The spin-polarized currents in the parallel and antiparallel configurations are examined to ascertain the cause of the MR ratio variation, as shown in Fig. 5b,c, respectively. The maximum MR ratios can be mainly attributed to very tiny spin-polarized current in the antiparallel configuration, as depicted in Fig. 5c (see Supplementary Note 3 for details). Additionally, the MR ratio dramatically declines as the bias voltage increases in Fig. 5a. However, MR ratios are always still up to 100% and satisfy the essential standards even when the bias voltage *V*_*SD*_ = 100 mV is applied. This is because the spin-polarized current in the parallel configuration is twice as large as that in the antiparallel configuration, as shown in Fig. 5b,c. We would like to note that an unfavorable non-ideality, such as contact resistance, could affect the performances in the real world^36,37^. As a result, it is fair to consider the extreme values in this study as the maximum of experimental observations due to the non-idealities.

In summary, we theoretically propose a gate-tunable spin-valve based on AGNR to produce a significant STT effect, allowing us to switch the magnetization of free layer at room temperature without the aid of an external magnetic field. It is found that the spin current density cannot surpass CSCD without applying gate voltage. Hopefully, with the help of the controlled gate, a tremendous spin current density of about 15 MA/cm^2^ is reached, far beyond the typical CSCD. It is suggested that the strength of exchange splitting may be as larger as possible with a gate voltage around 450 mV. The proposed spin-valve also allows for the achievement of ultralow writing power. The MR ratios are always up to 100% and meet the requirements in reading mode of MRAMs. These findings may pave the way for spin logic devices based on 2D materials to become feasible.

## Methods

### Two-dimensional Dirac Hamiltonian

The model Hamiltonian of the proposed system is given by

1 $$H={v}_{F}*\left(\widehat{\sigma }\cdot\widehat{p}\right)+V\left(x\right)-\xi {\Delta }_{ex},$$

where ${v}_{F}$ is the Fermi velocity, $\widehat{\sigma }$ is the vector of Pauli matrices, $\widehat{p}=\left({p}_{x}, { p}_{y}\right)$ is the in-plane momentum operator, $V\left(x\right)$ is the potential barrier, and ${\Delta }_{ex}$ is the exchange splitting energy induced by the magnetization of ferromagnetic lead. Spin-up (spin-down) index is denoted by $\xi =+1$($-1$).

### Landauer–Büttiker formalism

In Landauer-Büttiker formalism^38^, the spin currents with the different types of electrons injecting and leaving the system are given by

2 $${I}_{ij}=\frac{e}{h}{\int }_{-\infty }^{\infty }{T}_{ij}\left[{f}_{S}\left(E-{\mu }_{S}\right)-{f}_{D}\left(E-{\mu }_{D}\right)\right]dE,$$

where $h$ is the Plank constant, *e* is the electron charge, ${T}_{ij}$ is the transmission, and $f_{{S\left( D \right)}} = \left\{ {1 + exp\left[ {\left( {E - \mu _{{S\left( D \right)}} } \right)/k_{B} T} \right]} \right\}^{{ - 1}}$ is the Fermi–Dirac function with ${\mu }_{S(D)}.$ The detailed calculation of transmission is discussed in Supplementary Note 1.

### Spin transfer torque and spin current density

Spin transfer torque $\Gamma$ can be expressed as the following equation^24,25^

3 $$\Gamma = \frac{\hbar }{2e}J_{sp},$$

where $\boldsymbol{\hslash }$ is reduced Planck’s constant, and $e$ is the electron charge. The spin current density ${J}_{sp}={J}_{up}-{J}_{down}$ is defined, where ${J}_{up}={J}_{\uparrow \uparrow }+{J}_{\downarrow \uparrow }$ and ${J}_{down}={J}_{\downarrow \downarrow }+{J}_{\uparrow \downarrow }$ are given respectively. STT is proportional to the spin current density, as shown in Eq. (3). Thus, we can realize the STT effect in terms of spin current density.

## Supplementary Information

Supplementary Information.

## Data Availability

The data that support the findings of this study are available upon reasonable request from the corresponding author.
