# Supplementary material for: High spin current density in gate-tunable spin-valves based on graphene nanoribbons
Source: Sci Rep. 2023 Jun 7;13:9234. doi: 10.1038/s41598-023-36478-6 (PMC10247789; doi:10.1038/s41598-023-36478-6)
Supplement: Supplementary file 1 — Supplementary Information. [file 41598_2023_36478_MOESM1_ESM.docx]

**Supplementary information for**

**“High spin current density in gate-tunable spin valves based on graphene nanoribbons”**

**Chun-Pu Wang, Shih-Hung Cheng, and Wen-Jeng Hsueh***

Nanomagnetism Group, Department of Engineering Science, National Taiwan University

**Supplementary Note 1: Transfer matrix method for calculating transmission**

To calculate the transmission of spin currents passing through the entire system, the standard transfer-matrix method is applied on each segment of the device. Transfer matrix connects the wave functions of electrons through source (S) to drain (D) electrode, which can be expressed in the following equation

|  | $\left\{ \begin{matrix} a_{D}^{\rho} \\ b_{D}^{\rho} \end{matrix} \right\}=\Xi_{\rho\rho}\left\{ \begin{matrix} a_{S}^{\rho} \\ b_{S}^{\rho} \end{matrix} \right\}$, | (S.1) |
| --- | --- | --- |

where $\Xi_{\rho\rho}=N_{D\rho}^{-1}\prod_{j=1}^{m} {M_{j\rho}N}_{j\rho}^{-1}N_{S\rho}$, $\rho= \uparrow, \downarrow$ represents the spin orientations, *j* is the order of the segments, $a_{j}^{\rho}$ and $b_{j}^{\rho}$are the amplitude of the incident and reflected wave functions, and m is the total number of the layers located between source and drain. To be more specific, the element of matrices can be expressed as

|  | $M_{j\rho}=\left[ \begin{matrix} e^{ik_{xj}^{\rho}d_{j}} & {-e}^{ik_{xj}^{\rho}d_{j}} \\ {(k}_{xj}^{\rho}+ik_{y})e^{ik_{xj}^{\rho}d_{j}}/k_{j}^{\rho} & {-(k}_{xj}^{\rho}-ik_{y})e^{-ik_{xj}^{\rho}d_{j}}/k_{j}^{\rho} \end{matrix} \right],$ | (S.2) |
| --- | --- | --- |
|  | $N_{j\rho}=\left[ \begin{matrix} 1 & 1 \\ {(k}_{xj}^{\rho}+ik_{y})/k_{j}^{\rho} & {-(k}_{xj}^{\rho}-ik_{y})/k_{j}^{\rho} \end{matrix} \right].$ | (S.3) |

Note that $k_{y}$ are components of wave vector $k$, and $d_{j}$ is the length of the j^th^ layer.

The transmission can be therefore calculated by

|  | $T_{\rho\rho}=1-\left\vert\frac{\Xi_{21}}{\Xi_{22}} \right\vert^{2},$ | (S.4) |
| --- | --- | --- |

where $R_{\rho\rho}=\left| \frac{\Xi_{21}}{\Xi_{22}} \right|^{2}$ is the reflectivity. In the AGNRs, the quantized transverse momentum is restricted by the edge state. The quantized transverse wave vector $\left| k_{y}\left( m \right) \right|=\frac{m\pi}{3W} ,$ where *m* is an integer, and *W* is the width of AGNR [1-3].

**Supplementary Note 2: Charge current and polarization in gated spin-valve**

The charge current $I_{ch}$ and the polarization as a function of bias voltage are depicted in Fig. S1. The trend of the charge current is the same as the power, and it demonstrates the negative differential resistance effect as well, as shown in Fig. S1a. The charge currents are nearly the same when writing. The polarization is defined as $I_{sp}/I_{ch}$, which owns the local maximum around bias voltage *V_SD_* = 320 mV, as shown in Fig. S1b. It is recommended that the exchange splitting energy be as large as feasible, which can generate high polarization as well as spin current for switching at the writing voltage.

**Supplementary Note 3: High MR performance: transmission analysis with** $\boldsymbol{V}_{\boldsymbol{SD}}\boldsymbol{=10}\mathbf{mV}$

The MR ratio has been defined as, MR = $\frac{I_{p}-I_{ap}}{I_{ap}}\times100\%$, where $I_{p}$ and $I_{ap}$ are the spin-polarized currents in the parallel and antiparallel configurations respectively. According to Landauer-Büttiker formalism [4], the spin-polarized currents will be influenced by transmission and Fermi-Dirac function. Therefore, the MR performance can be characterized by the transmission spectrum. The spin-dependent transmissions are illustrated in Fig. S2. In Fig. S2c and S2d, the transmission functions are restrained in the antiparallel configurations, which result in a high MR ratio in the proposed spin-valve.

**Supplementary Note 4:** **The spin current density affected by the channel length.**

For every bias voltage provided, the spin current density remains near the constant valve as the channel length increases in Fig. S3. To exceed the critical spin current density, the minimum *V_SD_* = 400 mV must be applied. Note that the gate voltage of 500 mV is applied. The band gap energy is set to 150 meV, while the exchange splitting energy is set to 80 meV.

**SUPPLEMENTARY FIGURES**


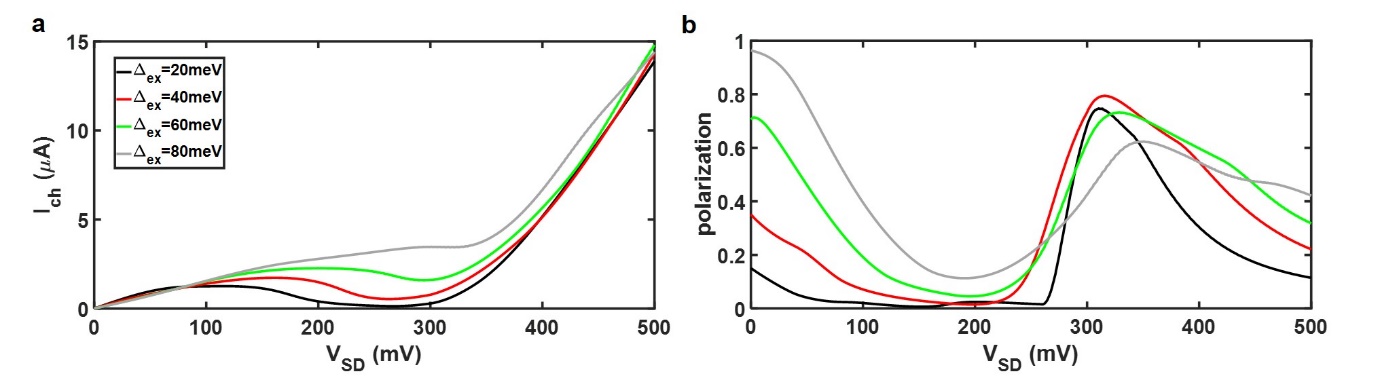


**Fig. S1 Charge current and polarization in gated spin-valves. a** Charge current and **b** polarization as a function of bias voltage with different exchange splitting energy. In **a** and **b**, the band gap energy is set to 150 meV, while gate voltage 450 mV, which generates the maximum spin current, is applied.


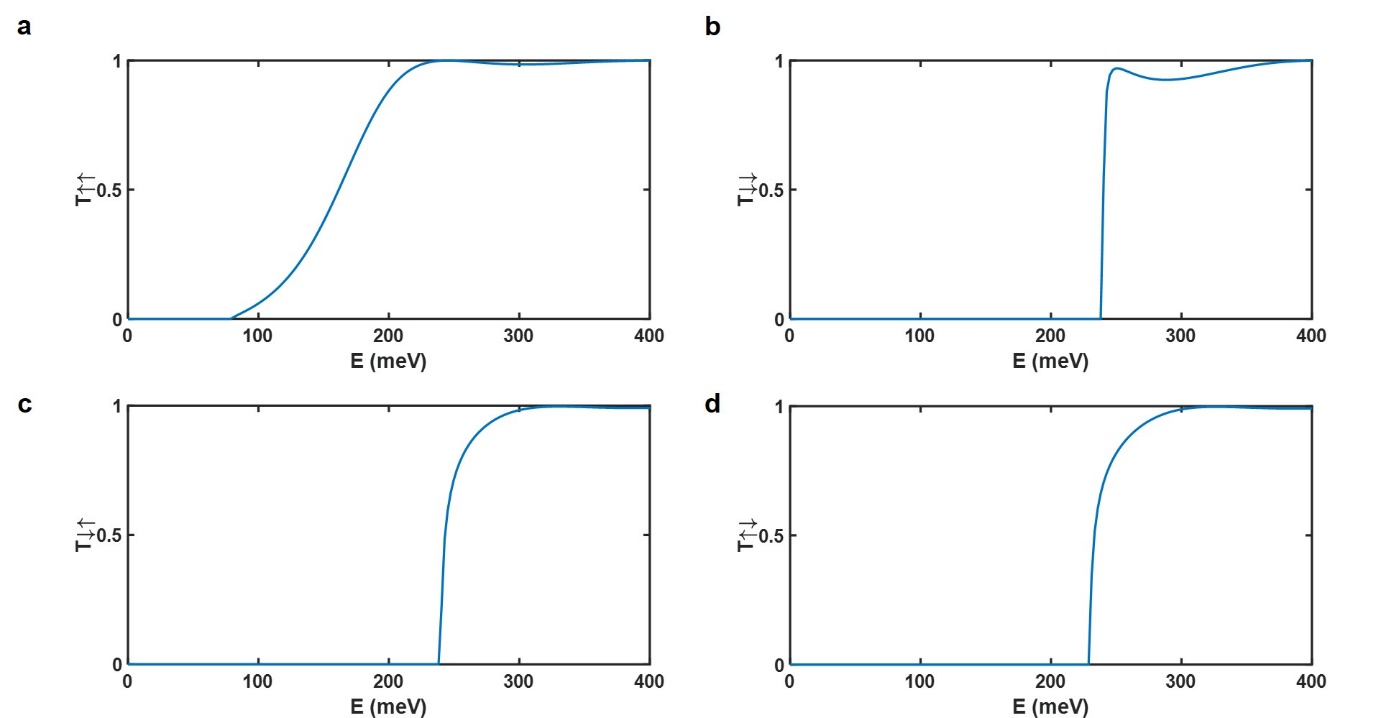


**Fig. S2 Spin-dependent transmission function versus electron energy with a small sensing bias voltage. a** *T_↑↑_*, **b** *T_↓↓_*, **c** *T_↓↑_*, and **d** *T_↑↓_* versus electron energy in the spin-valve.

**Fig. S3 The spin current density affected by the channel length.** As the channel length increase, the spin current density remains near the constant valve for every applied bias voltage.

**REFERENCES**

1. Munárriz, J. et al. Strong spin-dependent negative differential resistance in composite graphene superlattices. *Phys. Rev. B* **88**, 155423 (2013).
2. Gmitra, M. et al. Graphene on transition-metal dichalcogenides: A platform for proximity spin-orbit physics and optospintronics. *Phys. Rev. B* **92**, 155403 (2015).
3. Zollner, K. et al. Theory of proximity-induced exchange coupling in graphene on hBN/(Co, Ni). *Phys. Rev. B* **94**, 155441 (2016).
4. Blanter, Y. M. et al. Shot noise in mesoscopic conductors. *Phys. Rep.* **336**, 1-166 (2000).
